# Low-threshold spiking interneurons perform feedback inhibition in the lateral amygdala

**DOI:** 10.1007/s00429-020-02051-4

**Published:** 2020-03-06

**Authors:** Çağrı Temuçin Ünal, Bengi Ünal, M. McLean Bolton

**Affiliations:** 1 Department of Psychology, Comparative Cognition Laboratory, TED University, Ziya Gokalp Caddesi No. 48 06420, Kolej Cankaya, Ankara, Turkey; 2 Disorders of Neural Circuit Function, Max Planck Florida Institute for Neuroscience, Jupiter, FL 33458 USA

**Keywords:** Basolateral amygdala, Somatostatin, Parvalbumin, Interneuron, Low-threshold spiking, Fast-spiking, Feedback inhibition

## Abstract

Amygdala plays crucial roles in emotional learning. The lateral amygdala (LA) is the input station of the amygdala, where learning related plasticity occurs. The LA is cortical like in nature in terms of its cellular make up, composed of a majority of principal cells and a minority of interneurons with distinct subtypes defined by morphology, intrinsic electrophysiological properties and neurochemical expression profile. The specific functions served by LA interneuron subtypes remain elusive. This study aimed to elucidate the interneuron subtype mediating feedback inhibition. Electrophysiological evidence involving antidromic activation of recurrent LA circuitry via basolateral amygdala stimulation and paired recordings implicate low-threshold spiking interneurons in feedback inhibition. Recordings in somatostatin-cre animals crossed with tdtomato mice have revealed remarkable similarities between a subset of SOM+ interneurons and LTS interneurons. This study concludes that LTS interneurons, most of which are putatively SOM+, mediate feedback inhibition in the LA. Parallels with cortical areas and potential implications for information processing and plasticity are discussed.

## Introduction

Amygdala is a collection of different nuclei (Swanson and Petrovich 1998) that play cardinal functions in the acquisition and expression of fear responses (LeDoux 2000; Pape and Pare 2010; Gründermann and Lüthi 2015). The basolateral complex of the amygdala (BLA) and central amygdala (CeA) constitute the two main nuclei. The BLA is a collection of smaller cortex-like nuclei and is composed ~ 75% of glutamatergic principal neurons that relay information to the output station of the amygdala, the central amygdala (CeA) (McDonald 1992). These BLA glutamatergic principal neurons exhibit morphological and electrophysiological similarities with their cortical counterparts (Faber et al. 2001).

The remaining population (~ 25%) constitutes a neurochemically and electrophysiologically heterogeneous set of interneurons similar to that found in cortex (McDonald and Augustine 1993; Pare and Smith 1993; Spampanato et al. 2011; Bienvenu et al. 2012; McDonald and Augustine 2019) and are thought to play significant roles in determining the integration and plasticity of principal cell synaptic inputs and determining the dynamic range of their action potential firing output (Gaudreau and Pare 1996), thereby regulating information processing within the basolateral amygdala under normal and pathophysiological conditions (Letzkus et al. 2015; Fee et al. 2017; Krabbe et al. 2018).

The most salient parallels between BLA and other cortical regions with respect to their interneurons exist with respect to parvalbumin (PV) and somatostatin (SOM) positive interneurons. For instance, PV+ interneurons form perisomatic baskets or axoaxonic synapses on principal neurons and constitute around 50% of the total interneuron population (McDonald and Mascagni 2001; McDonald and Bettette 2001; Rainnie et al. 2006; Vereczki et al. 2016; Butler et al. 2018) and play significant roles in fear learning (Lucas et al. 2016). Remarkably, these PV+ interneurons exhibit a close correspondence in their electrophysiology to their cortical counterparts (Rainnie et al. 2006; Woodruff and Sah 2007). SOM positive interneurons constitute the other common interneuron population and they co-express markers such as neuropeptide Y (NPY) and calbindin, an expression profile similar to cortical SOM+ interneurons (McDonald and Mascagni 2002; Truitt et al. 2009). Similar to cortex, SOM+ interneurons selectively target the dendrites of BLA principal neurons (Muller et al. 2007).

However, there appears to be some interesting discrepancies. Calbindin positive/PV− interneurons which are putatively SOM+ have been shown to receive intensive extra BLA input in anatomical studies (Unal et al. 2014) while anatomical and physiological data implicate PV+ interneurons receiving a majority of their excitatory inputs from principal BLA neurons (Smith et al. 2000; Woodruff and Sah 2007; Unal et al. 2014; Spampanato et al. 2016). These findings implicate SOM+ interneurons in feedforward inhibition while PV + interneurons appear to mediate feedback inhibition. The opposite scenario is thought to occur in other cortical regions (Urban-Ciecko and Barth 2016; Yavorska and Wehr 2016). It is still not clear whether these discrepancies involve the entire basolateral complex or whether there are subnucleus specific exceptions.

Importantly, BLA is composed of multiple smaller subnuclei that display hodological and functional distinctions (McDonald 1998; Pare et al. 2004). Among these nuclei, the lateral amygdala (LA) is the input station of the amygdala and it is thought to be the critical site of plasticity for the acquisition of fear memories (LeDoux 2000). In the current study, we aimed to identify the interneuron type that mediates feedback inhibition focusing specifically to LA to investigate whether our observations will parallel the observations made in the BLA overall. We have found a proportion of low threshold spiking (LTS) interneurons to exhibit reciprocal connectivity with principal cells of the LA while fast-spiking (FS) interneuron inputs were not reciprocated by principal neurons. Using transgenic cre lines crossed with tdtomato mice, we tested whether SOM and PV interneurons correspond to the electrophysiologically defined interneurons in our initial experiments. We have found that SOM+ interneurons exhibiting the LTS profile were reciprocally connected with principal neurons. These findings suggest that a portion of LTS interneurons that are SOM+ mediate dback inhibition in the LA. Further experiments need to test to what extend this architecture extends to other BLA nuclei.

## Materials and methods

Procedures were approved by the Institutional Animal Care and Use Committee of Max Planck Florida Institute, in compliance with the Guide for the Care and Use of Laboratory Animals (DHHS).

### Animals

All experiments were done in adult male mice (2–4 months old). C57BL/6J mice were used for the initial experiments. For targeted somatostatin interneuron recordings, SOM-Cre (B6N.Cg-Ssttm2.1(cre)Zjh/J) and tdTomato (B6.Cg-Gt(ROSA)26Sortm9(CAG-tdTomato)Hze/J) crosses were used. For targeted parvalbumin interneuron recordings, PV-Cre (B6N.129P2-Pvalbtm1(cre)Arbr/J) and tdTomato (B6.Cg-Gt(ROSA)26Sortm9(CAG-tdTomato)Hze/J) crosses were used. These mice were obtained from Jax Laboratories (Bar Harbor, ME). All mice were housed in AAALAC accredited animal facility with food and water ad libitum.

### Slice preparation

Mice (PD 60–PD 120) were deeply anesthetized with isoflurane and transcardially perfused with 10 ml of an ice cold solution containing (in mM) 124 choline chloride, 2.5 KCl, 1.2 NaH_2_PO_4_, 3.3 MgCl_2_ 26 NaHCO_3_, 10 glucose, and 0.5 CaCl_2_. The brains were then removed from the skull and blocks containing the amygdala were prepared. Subsequently, 300 μm thick coronal sections containing the LA were obtained with a vibrating microtome using the same ice cold solution. The slices were then transferred to a holding chamber filled with an oxygenated (with 95% O_2_, 5% CO_2_) artificial cerebrospinal fluid (ACSF) solution containing (in mM) 115 NaCl, 3 KCl, 1.25 NaH_2_PO_4_, 26 NaHCO_3_, 10 glucose, 1 MgCl_2_, 2 CaCl_2_, 5 sodium ascorbate, 3 sodium pyruvate and 2 thiourea at 32 °C. Following ~ 15 min of incubation at 32 °C, the slices were transferred into another holding chamber containing the same solution at room temperature (~ 22 °C).

### Electrophysiological recordings and analysis

One hour or later (max 4 h), one slice was transferred to a custom made recording chamber superfused with oxygenated ACSF (3–5 ml/min). LA neurons were visualized with an Olympus BX51WI (Center Valley, PA) microscope, equipped with infrared differential contrast optics. Under visual guidance, we obtained whole-cell recordings of LA neurons using pipettes (3–6 MΩ) pulled from borosilicate glass capillaries and filled with a solution containing (in mM): 145 potassium gluconate, 5 NaCl, 10 HEPES, 0.5 EGTA, 4 MgATP, 0.3 Na_2_GTP, for current clamp recordings and 120 Cs methanesulfonate, 8 NaCl, 15 CsCl, 10 TEACl, 10 HEPES, 0.5 EGTA, 10 QX-314, 4 MgATP and 0.3 Na_2_GTP for voltage clamp recordings. The intracellular solutions were adjusted to a pH around 7.25 ± 0.03 and the osmolarity was adjusted to 290 mOsm ± 5. In cases where the recording technique required a morphological analysis of the neurons, biocytin (0.2%) was added into the intracellular solution. Current-clamp recordings were obtained with an Axoclamp 700B amplifier and digitized at 10 kHz with a Digidata 1440A interface (Molecular Devices, Palo Alto, CA). Data acquisition ensued 5–10 min after whole cell access. During experiments, access resistances were monitored before the onset of each electrophysiological protocol. Cases where the access resistances exceeded 20 MΩ were discarded from analysis.

### LA circuitry analysis

**Fig. 1:**
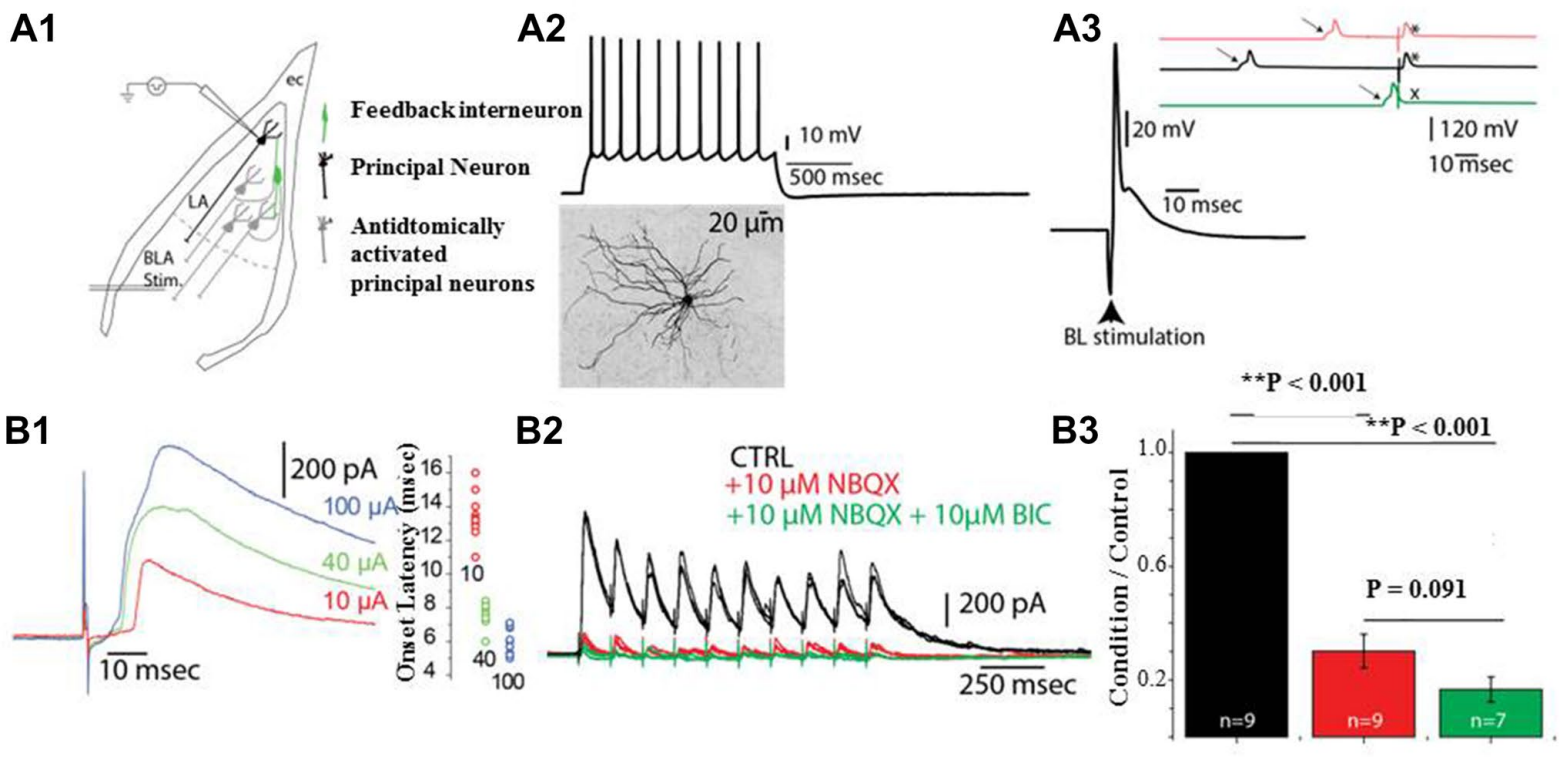
Intranuclear inhibition in the LA. **a** Experimental protocol (**a1**), action potential discharge in an LA neuron in response to intracellular current injections and morphological features (**a2**), and an antidromic spike in the same neuron as a result of BL stimulation. Inset, three individual traces from a collision test (arrow, intracellular current evoked spike; asterisk, antidromic spike; x, collision) (**a3**). **b** Voltage clamp recordings (V_hold_ = 0 mV) reveal BL evoked polysynaptic IPSCs with a jitter in delays with increasing BL stimulation intensities (**b1**), and pharmacological profiling, revealing a high sensitivity to glutamatergic receptor signaling (**b2 **and **b3**). Values in color in **b1** represent the BL stimulation intensity (**b1**). Representative data of a neuron recorded under different pharmacological conditions (**b2**). Quantification of pharmacological data (**b3**). Note that the majority of the inhibitory response disappears after the addition of AMPA-receptor blocker NBQX (10 µM)

The investigation of intrinsic circuitry was multifaceted and involved various paired recording and electrical stimulation procedures. Preliminary analysis of intra-LA inhibition involved recording the responses of LA neurons to the stimulation of the basolateral amygdala (BL). In these experiments, recordings of LA neurons were done from a region corresponding to 1 to 1.4 mm posterior to Bregma. The stimulating electrodes were placed to a depth corresponding to 4–4.5 mm from the brain surface at the center of the BL (3–3.75 mm lateral to the midline). We typically used 100 μA stimulation intensity unless otherwise indicated. Since information transfer between LA and BL is largely unidirectional, directly stimulating the BL would recruit descending LA fibers, antidromically stimulating the LA neurons (see Fig. 1a1 for a schematic representation). In these cases, the lidocaine derivative QX-314 (10 µM) was added to the cesium based intracellular solution to block action currents in the recorded neurons that were kept at 0 mV in voltage clamp to isolate the polysynaptic IPSCs that originate from local LA interneurons stimulated by other LA cells. In pilot experiments, a potassium-based intracellular solution was used instead of a QX-314 added cesium-based intracellular solution. In these cases, BL stimulation unequivocally led to action potentials LA neurons (not shown). Other pharmacological procedures were used to ascertain the recurrent nature of these evoked IPSCs (see results). These experiments were done in non-transgenic mice, somatostatin- and parvalbumin-tomato crosses, and GAD-GFPs to isolate the specific interneuron type that mediates recurrent inhibition.

In paired recordings, the potential presynaptic cell was stimulated at 50 Hz for 500 ms every 20 s and 15–20 traces in the potential postsynaptic neuron was averaged both for ~ − 70 and ~ − 55 mV in current clamp for visualizing possible unitary EPSPs or unitary IPSPs (or polysynaptic IPSPs) respectively. In another set of paired recording experiments, the potential postsynaptic neurons were tested only ~ − 70 mV (for EPSPs only) in the presence of bicuculline (10 µM). In cases where a monosynaptic connection was observed, a protocol involving 20 Hz stimulation of the presynaptic neuron for 500 ms (every 20 s) was carried out and 40 to 50 such traces were acquired. All these tests were done with potassium based intracellular solutions in a bidirectional manner in current clamp. A typical strategy involved patching one neuron and sequentially recording other neurons with another pipette 2 for investigating the connectivities.

Rise time constants of synaptic events were calculated by fitting a single exponential between 10 and 90% of the maximal response. For paired recordings among monosynaptically connected pairs, the formal analysis was restricted to the last evoked synaptic event evoked by the post-tetanic stimulation. For polysynaptic pairs, IPSPs were detected using the event detection tool in clampfit. All available synaptic events for a particular pair (averages of the last evoked postsynaptic potentials in monosynaptically connected pairs and all detected IPSPs that could be detected using clampfit threshold detection) were averaged to obtain a single rise time constant value.

The distance between pairs of neurons tested ranged from 10 to 200 microns in all cases.

### Definition of feedback vs. non-feedback interneurons

Among cells that satisfied the electrophysiological criteria for interneurons (i.e. a non-regular firing pattern and producing IPSPs in a postsynaptic neuron), those where at least 4 potential principal neuron presynaptic partners were probed were classified either as feedback or non-feedback interneurons depending on whether they received feedback connections from principal neurons (Fig. 4). This number is based on connectivity ratios between principal neurons and feedback interneurons in the BLA (Woodruff and Sah 2007) and other cortical areas (Yavorska and Wehr 2016).

### Intrinsic physiology of interneurons

Neurons were kept at − 70 mV (± 3 mV) in current clamp with bias current injections. Graded square pulses (− 100 to 200 pA in 20 pA increments) were applied every 5 s for 1.5 s.

Input resistances of neurons were estimated from the linear portion of the voltage–current (*V*–*I*) curve. The time constants were measured by fitting an exponential to the voltage pulse starting with the offset of the current injection to return to baseline.

Action potential threshold was considered as the point where the rising slope of voltage exceeded 10 mV/ms at rheobase.

Other firing properties measured were the maximum firing frequency and adaptation ratio. The former was defined as the maximum frequency reached within the range of square pulses applied from − 70 mV. The latter was calculated at half maximal firing as “interspike interval (ISI) for the last two spikes/ISI for the first two spikes”.

### Biocytin visualization and immunohistochemistry

Following recordings with biocytin in the pipette (0.2%), slices were transferred to 4% paraformaldehyde for at least 24 h for fixation. For biocytin visualization, slices were incubated in 1:200 Cy3-conjugated streptavidin or Texas Red conjugated streptavidin (Invitrogen, Carlsbad, CA; Jackson ImmunoResearch Laboratories, West Grove PA) for 20–24 h. Slices were then transferred to a slide and mounted with Vectashield wet mounting medium (Vector Labs Inc., Burlingame CA).

Slices were fixed in 4% PFA in 0.01 M PBS overnight. Following PBS washes and permeabilization with 1% Na-Borohydride, 10% Methanol and 3% H_2_O_2_ in PBS, slices were incubated in 5% BSA, 5% Triton X-100 and 1:400 Streptavidin-AlexaFluor 488/594 conjugate overnight. For PV staining, mice were perfused with 4% PFA and brains kept in 4% PFA overnight at 4 ℃. 50 µm sections were collected. After the same cleaning and permeabilization steps as mentioned above, blocking was done in a cocktail of 10% NDS, 2% BSA, 5% Triton X-100 overnight at 4 ℃. Sections were then incubated in 1:1000 Goat-Anti-PV antibody in PBS for 48–72 h at 4 ℃. Sections were transferred into 1:400 Donkey-Anti-Goat AlexaFluor488 for 4–6 h and then rinsed in PBS prior to mounting. The same procedures were applied for somatostatin staining where the Goat-Anti-PV antibody was replaced with 1:1000 Goat-Anti-Somatostatin antibody.

### Confocal laser scanning microscopy and morphological reconstructions

Digital images of biocytin-filled neurons and immunostained sections were acquired with a Zeiss LSM780 confocal microscope. The digital images were used for the 3-D reconstruction of neurons using the NeurolucidaTM Software (MBF Bioscience, VT, USA). For purposes of clarity, some biocytin filled neuron images are inverted versions of a gray scale image.

### Statistical analysis of the data

All data were analyzed using Origin 7.0 (Northampton, MA, USA) and SPSS (Chicago, IL).

## Results

### BL Stimulation and inhibition in LA neurons

The following set of experiments was conducted to investigate whether the current slice preparation method preserves the inhibitory circuitry within the LA. When LA principal neurons were recorded with a potassium based internal solution, BL nucleus stimulation resulted in antidromic spikes in 5/22 (~ 20%, 22 cells obtained from 4 animals) of LA neurons, consistent with previous experiments (Samson et al. 2003; Fig. 1a3). Compared to synaptically evoked spikes, these spikes were insensitive to NMDA and AMPA receptor antagonists, had a sudden onset following stimulation, exhibited a fixed latency, collided with intracellular current evoked spikes and arose from the baseline (Fig. 1a3). In these experiments, the identity of principal neurons were mainly verified through intracellular current injections to evoke spikes, which exhibit adaptation in principal neurons (Fig. 1a2; Washburn and Moises 1992; Faber et al. 2001) and morphological verifications (Fig. 1a2, bottom).

Next, we sought to determine if the recruitment of principal projection neurons result in polysynaptic inhibitory events, IPSCs in this case, in other principal neurons. For this purpose, cesium-based intracellular solutions supplemented with the lidocaine derivative QX-314 (10 µM) were used for recordings. This approach assured that the postsynaptic voltage gated sodium channels are blocked and recorded neurons were not directly affected by the BL stimulation. Responses at 0 mV were considered to be purely inhibitory based on the reversal potential of glutamatergic transmission under our experimental conditions (see methods). These responses did not have a fixed latency, displayed earlier onsets with stronger stimuli (Fig. 1b1) and were largely inhibited by glutamatergic receptor blockade (Fig. 1b2), suggesting that BL interneuron axons (Bienvenu et al. 2012) contribution to the IPSCs we observed are minimal. Furthermore, the magnitude of inhibition (paired *t *test, *p* < 0.05) with glutamatergic receptor blocker reached a peak, and GABA-A receptor inhibition did not result in an extra blockade (Fig. 1b2 and b3; paired *t* test, *p* > 0.05; 11 neurons recorded from 4 animals). These data suggest that the IPSC seen under these conditions were polysynaptic in nature and result due to intranuclear recruitment of interneurons. Nonetheless, the polysynaptic recruitment might also result from the polysynaptic activation of BL interneurons (Bienvenu et al. 2012). Hence, the current slice approach is permissive, but not immune to problems, for studying the mechanisms of intranuclear inhibition.

### Inhibition revealed with LA principal neuron pairs

The following sets of experiments involving paired recordings were conducted to unequivocally illustrate that the IPSCs observed in the previous experiment stem from local LA circuitry.

**Fig. 2:**
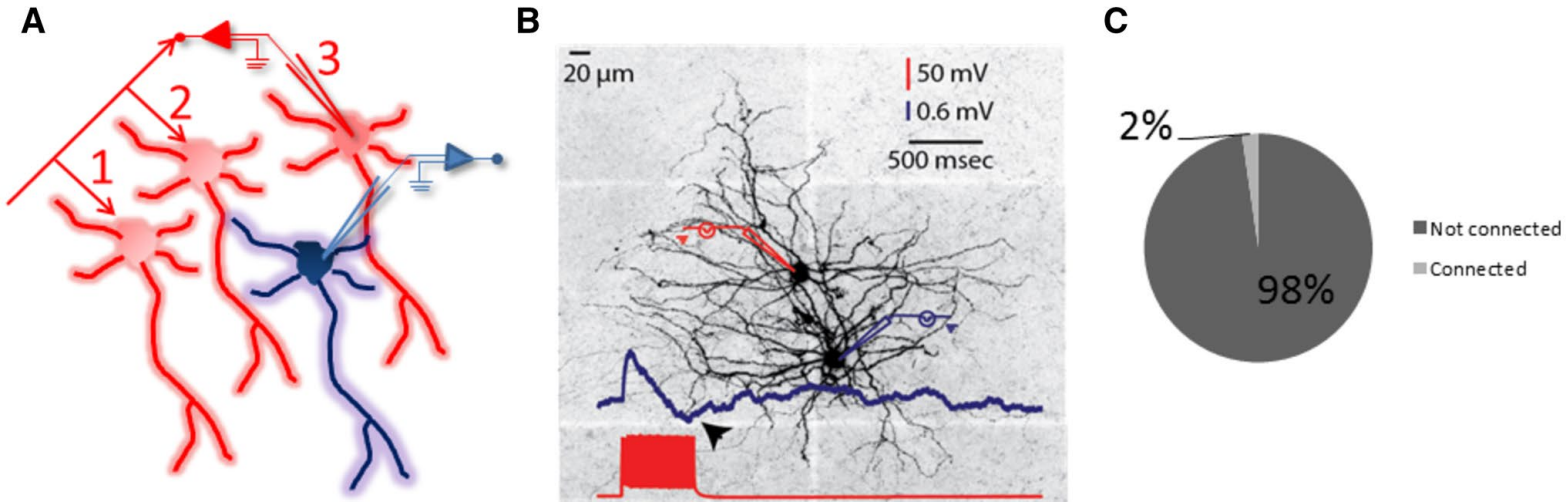
Monosynaptic connectivity among LA principal neurons. **a** An illustration for serial connectivity search. In this example, an LA neuron is held throughout the recording (blue neuron) and synaptic partners are searched (red neurons) in a sequential manner. The numbers in the illustration indicate the order with which synaptic partners are searched. **b** A synaptically connected pair in a unidirectional manner. 50 Hz stimulation of the presynaptic neuron (red neuron) triggers a barrage of EPSPs followed by a hyperpolarization (arrow), putatively a polysynaptic IPSP barrage. **c** Pie chart illustrating the ratio of connected pairs with and without bicuculline combined (4 out of 211 pairs; ~ 1.89% combined)

We obtained 58 principal neuron pairs from 10 animals with drug free ACSF using K-gluconate based intracellular solutions. Please refer to “LA circuitry analysis” in the methods section for a description of paired recording strategies (refer to Fig. 2a for a brief description). We found a unidirectional monosynaptic connection in one of these cases (~ 1.72%) (Fig. 2b, c). In this case, the monosynaptic EPSP barrage was followed by a hyperpolarization, possibly a polysynaptic inhibition.

Because tonic inhibition present in the LA (Marowsky et al. 2012) has the potential to mask small EPSPs, we carried out additional experiments with bicuculline (10 µM) in the bath. An additional 153 pairs were recorded 15 animals where 3 unidirectional monosynaptic connections were observed (1.96%). Hence, 211 pairs were recorded in total and only 4 monosynaptic connections were observed, corresponding to a ~ 1.89% connectivity rate (not shown).

**Fig. 3:**
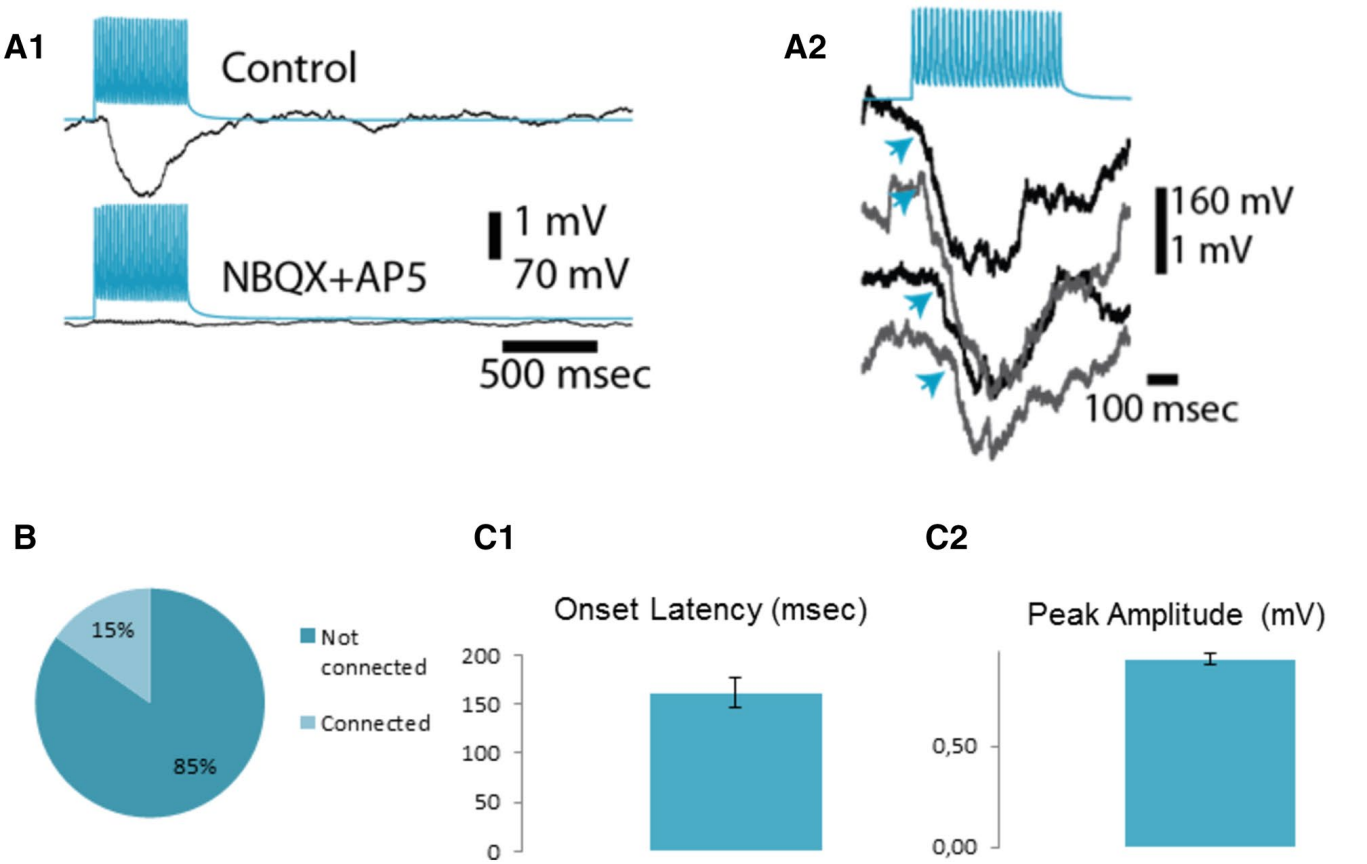
Polysynaptic inhibition observed in LA principal neuron pairs. **a** Recordings of polysynaptic IPSPs in LA principal neuron pairs. Postsynaptic inhibitory responses (black traces) to a 500 ms, 50 Hz stimulation of the other LA principal neuron (blue traces) (average of 50 traces; **a1** top). The postsynaptic response dissipating as a result of bath application of CNQX (10 μM) and AP-5 (50 μM) (**a1**, bottom). Representative individual traces demonstrating the variability in the onset of polysynaptic IPSP barrages (**a2**). **b** Incidence of polysynaptic connections among principal LA neurons as illustrated by a bar chart. **c** Properties of polysynaptic IPSP barrages. Onset latencies (**c1**) and peak amplitudes (**c2**)

In contrast to the scarcity of monosynaptic connections (4 out of 211, groups combined), the incidence of polysynaptic inhibition was relatively high (9 out of 59 pairs tested corresponding to ~ 15.25%; Fig. 3b). As mentioned before, in one case, we observed a monosynaptic EPSP barrage before the occurrence of polysynaptic IPSPs. This pair was removed from the analysis of amplitude and onset analysis of polysynaptic IPSPs. The average peak IPSP amplitude for all traces acquired was 0.93 ± 0.03 mV (data from 8 pairs, 160 traces). Evidence for the polysynaptic nature of these connections comes from the irregularity of IPSP onsets in the postsynaptic neuron (161 ± 15 ms; Fig. 3a2) and the sensitivity of the postsynaptic IPSPs to glutamatergic receptor blockers (Fig. 3a1, bottom traces). The rise constants (Fig. 5d) of polysynaptic IPSPs were relatively slow (from 6 to 11 ms), which guided us in later experiments (see below) to putatively determine the interneuron mediating these effects.

In short, these experiments revealed that polysynaptic IPSPs in the LA can be shown with paired recordings and the kinetics of these IPSPs have the potential to be utilized in finding the interneuron that mediates feedback inhibition.

### Interneuron-Principal neuron pairs: differences between feedback and non-feedback interneurons

**Fig. 4:**
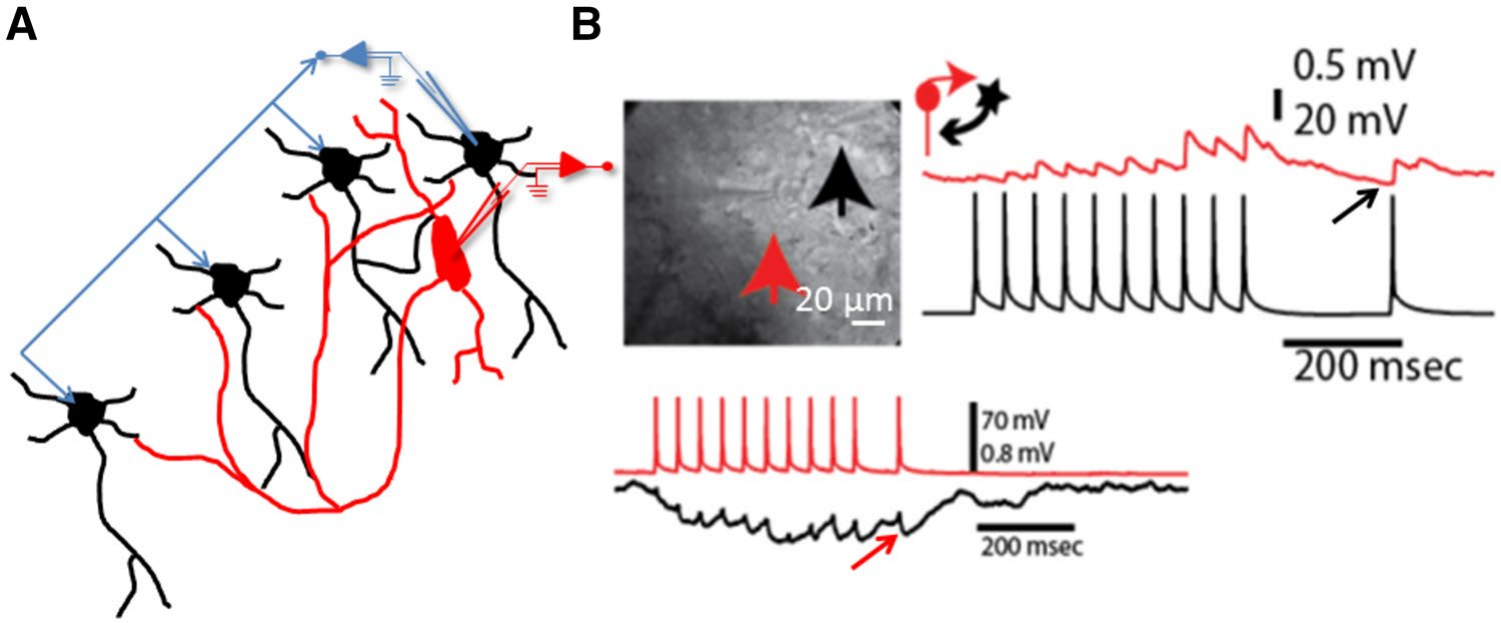
Identification of feedback interneurons. **a** An illustration of a serial paired recordings which involves recording of an interneuron (shown in red) while probing at least four principal potential synaptic partners (shown in black) in a bidirectional manner. **b** A representative recording from a feedback interneuron bidirectionally connected to its principal neuron synaptic partner. The image in the inset illustrates the distance between recorded neurons. For ease of illustration, the recorded neurons are shown with arrows colored correspondingly. Averaged electrophysiological traces from the recording showing connectivity in principal neuron—> feedback interneuron (top traces) and feedback interneuron—> principal neuron (bottom traces) direction

**Fig. 5:**
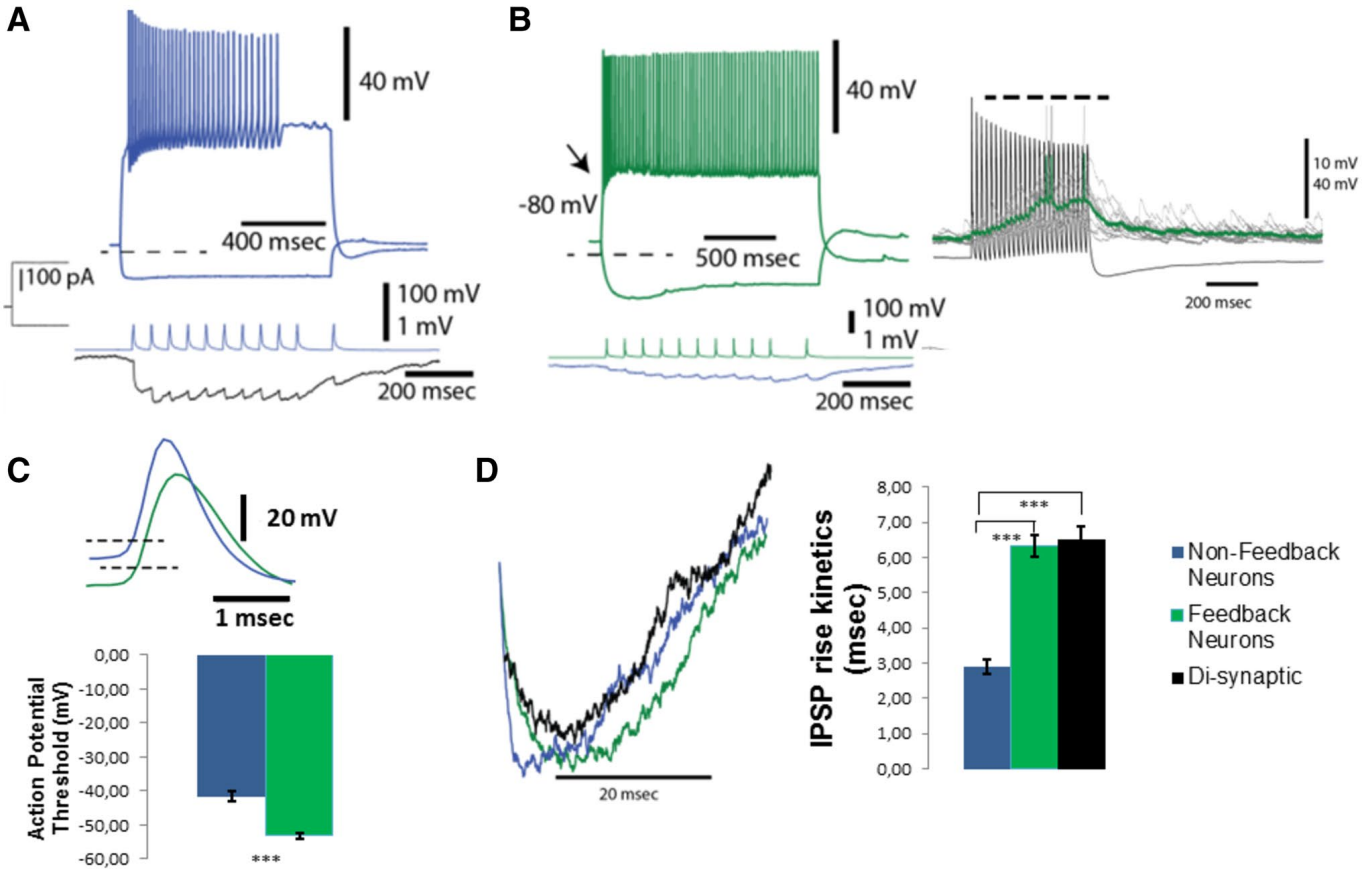
Feedback interneurons can be electrophysiologically defined as low-threshold spiking (LTS) interneurons. **a** Firing pattern of a nonfeedback interneuron (top panel) and IPSPs it elicits in a principal neuron (bottom panel, black trace, average of 20 raw traces). **b** Firing pattern of a feedback (LTS) interneuron (left, top panel) and IPSPs it elicits in a principal neuron (bottom panel, black trace, average of 20 raw traces). Black arrow on the top panel indicated low-threshold spikes riding on a putative calcium plateau. On the right, responsiveness of the LTS interneuron to principal cell firing is illustrated. EPSP evoked spikes in the LTS interneuron are truncated. The dashed lines in A and B indicate the respective membrane potential values. **c** Comparison of representative action potentials in a feedback (green trace) and a non-feedback (blue trace) neuron. Note the difference in the width and threshold of the action potentials. Bar graph illustrating the difference in the action potential threshold of feedback and non-feedback interneurons (the same color coding as in the representative traces. *** indicates a significant difference with *p* < 0.000 (C2). **d** Representative traces illustrating the similarity between the rise kinetics in LTS elicited IPSPs to those IPSPs seen during polysynaptic inhibition (color coding is the same for feedback and non-feedback interneuron elicited IPSPs; black trace indicates an IPSP elicited as a result of polysynaptic input). Bar graph on the right summarizes statistical comparisons. *** indicates a significant difference with *p* < 0.000

Experiments involving principal neuron–interneuron paired recordings were done to directly observe the interneuron type fulfilling the feedback inhibition function. Two things constitute the defining criteria of feedback interneurons: 1—to receive excitatory inputs from the local collaterals of principal neurons; 2—to inhibit the principal neurons. To determine the complete profile of feedback interneurons within the LA microcircuitry, a comparison with non-feedback interneurons is necessary. To formulate a strong definition for non-feedback neurons, we considered any interneuron not connected to its 4 neighboring principal neurons as a “non-feedback” interneuron (Woodruff and Sah 2007). Other interneurons where a sufficient number of potential presynaptic partners were not probed were not taken into consideration. We mainly used a search approach until finding interneurons (Popescu and Pare 2010) and connections where at least 4 principal neurons were serially recorded and tested for bi-directional connectivity while an interneuron was being recorded (Fig. 4). In these experiments, we recorded 14 interneurons from 8 animals that satisfied the abovementioned criteria for differentiating “feedback” and “nonfeedback” interneurons. These neurons were easily classified as interneurons due to their electrophysiological dissimilarity to principal neurons (Fig. 5a, b, top traces) and the unitary IPSPs they generated in their postsynaptic principal neuron partners (20/35 for “feedback” interneurons; 25/32 for “non-feedback” interneurons; Fig. 5a, b, bottom traces). For each interneuron, we probed at least 4 potential excitatory presynaptic neurons. For seven of these interneurons, no principal excitatory cell synaptic partner was found and therefore these neurons are tentatively referred as non-feedback interneurons (none of the 32 principal neurons tested for synaptic connectivity were found to trigger uEPSPs in these neurons). Interestingly, these “non-feedback” interneurons could be classified as conventional fast-spiking or stuttering fast-spiking interneurons based on their fast discharge rates and short-duration action potentials (Fig. 5). For the remaining 7 interneurons, we probed 35 potential excitatory presynaptic partners from principal cells. Overall, 13 out of the 35 principal neurons (~ 37%) were found to provide unitary EPSPs (0.22 ± 0.03 mV) to these “feedback” interneurons (Fig. 5b, right). Unlike their fast-spiking counterparts, these neurons had much lower action potentials thresholds (Fig. 5c; *t* (12) = 7.252, *p* < 0.0001), wider action potentials (Fig. 5b; *t* (12) = 4.083, *p* = 0.0015), they exhibited a voltage sag, indicative of an h-current (Fig. 5b), and they exhibited a continuum of spike frequency adaptation. Based on the fact that action potential threshold is the factor that clearly distinguishes feedback interneurons from non-feedback interneurons (Fig. 5c1), feedback interneurons are from now on referred as low-threshold spiking (LTS) feedback interneurons.

The average paired-pulse ratio (EPSP2/EPSP1 for two action potentials separated 50 ms apart) of these unitary EPSPs were (1.28 ± 0.14) (not shown). This facilitating pattern was also evident with stimulus trains of stimulations that ranged from 20 to 50 Hz in our connection search protocols (Fig. 4b, right). The rise and decay time constants for the uEPSPs were 4.43 ± 0.76 ms and 15.43 ± 6.47 ms, respectively.

Conversely, IPSP rise kinetics between di-synaptic IPSPs and LTS feedback interneuron firing evoked IPSPs exhibited striking similarities (H (4.15, *p* < 0.0001, see Fig. 5d for pairwise comparisons). This reinforced our thinking that the interneuron mediating the polysynaptic inhibition is the LTS interneuron (Fig. 5d). Specifically, the rise time constants of both disynaptic IPSPs and LTS evoked IPSPs ranged between 6–11 ms and largely overlapped (6.50 ± 0.39 ms for polysynaptic inhibition; 6.33 ± 0.35 ms for LTS evoked unitary IPSPs) while nonfeedback interneuron evoked IPSP rise times had a range of 2–4 ms (2.89 ± 0.20 ms) and exhibited no overlap with former IPSPs.

### Further characterization of LTS feedback interneurons: Use of SOM- and PV-cre tomato animals

To further characterize the interneuron type involved in feedback inhibition, we used SOM-Tomato and PV-Tomato animals in initial experiments to characterize their intrinsic physiology and synaptic connectivity (both paired recordings and BL-evoked EPSPs) and contrast these findings to the abovementioned results.

**Fig. 6:**
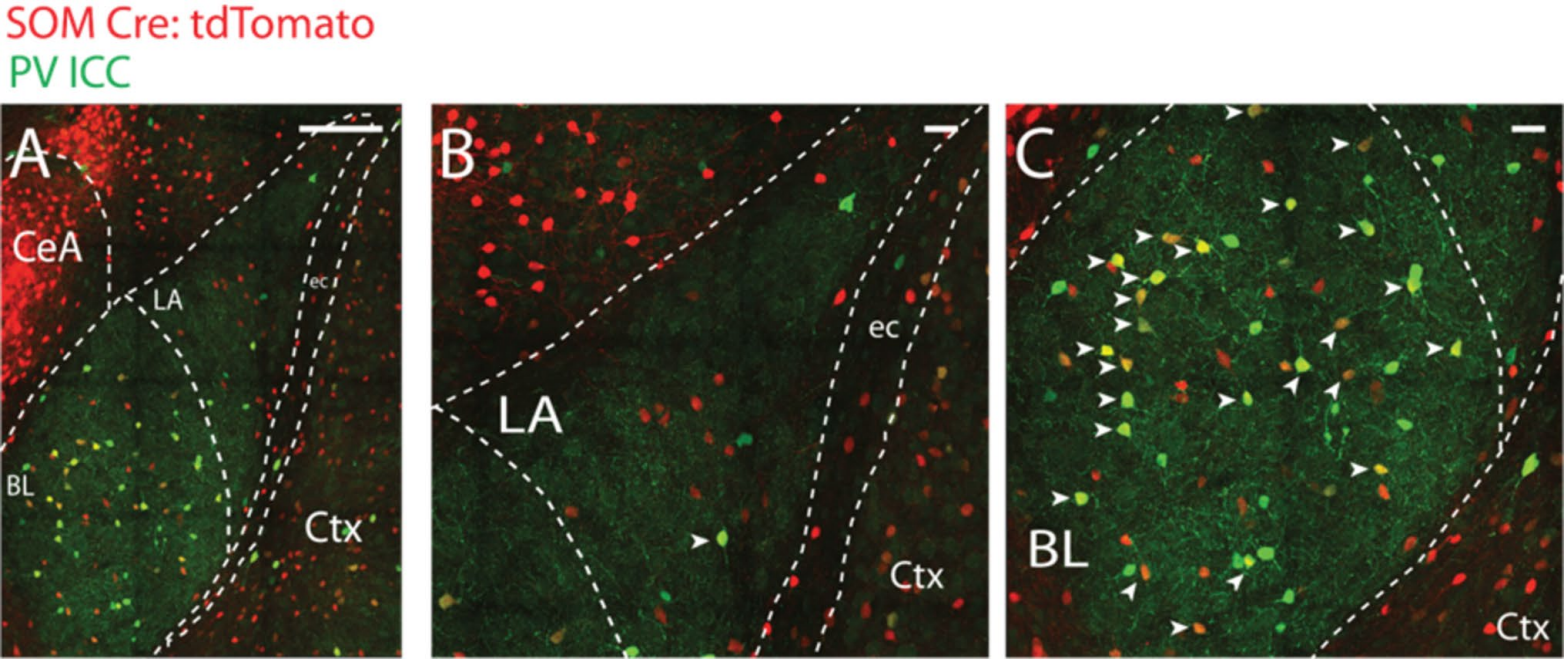
Somatostatin (SOM)-tomato positive and Parvalbumin (PV) immune-positive interneurons constitute different populations of neurons within the LA but not within the BL. **a** Lower magnification image of the BLA complex illustrating PV immunoreactivity (green) in SOM-Cre:tdTomato mouse slice. **b** Magnified view of the LA from *A*. Note the absence of co-labeling. **C** Magnified view of the BL from *A*. Note the abundance of PV and SOM co-expression in neurons. SOM-tomato/PV double positive neurons constituted 3% and 53% of LA and BL SOM-tomato neurons in the LA and BL respectively

First, we intended to determine the overlap of PV and SOM within the LA, the main interest of the current project. To do that, we ran immunocytochemistry for PV in 3 SOM tomato animals. While substantial overlap was observed within the BL with 53% of SOM tomato neurons co-expressing PV (96 SOM tomato neurons counted from LA sections belonging to three mice), there was minimal overlap within the LA with only 3% of SOM tomato neurons exhibiting PV immunoreactivity (135 SOM tomato neurons counted from LA sections belonging to three mice) (Fig. 6). The overlap seen in the PV neurons in the BL might reflect transient developmental expression of SOM in PV neurons (Hu et al. 2013) as SOM protein levels are observed to decrease as maturation proceeds (Forloni et al. 1990; Papadopoulos et al. 1993).

**Fig. 7:**
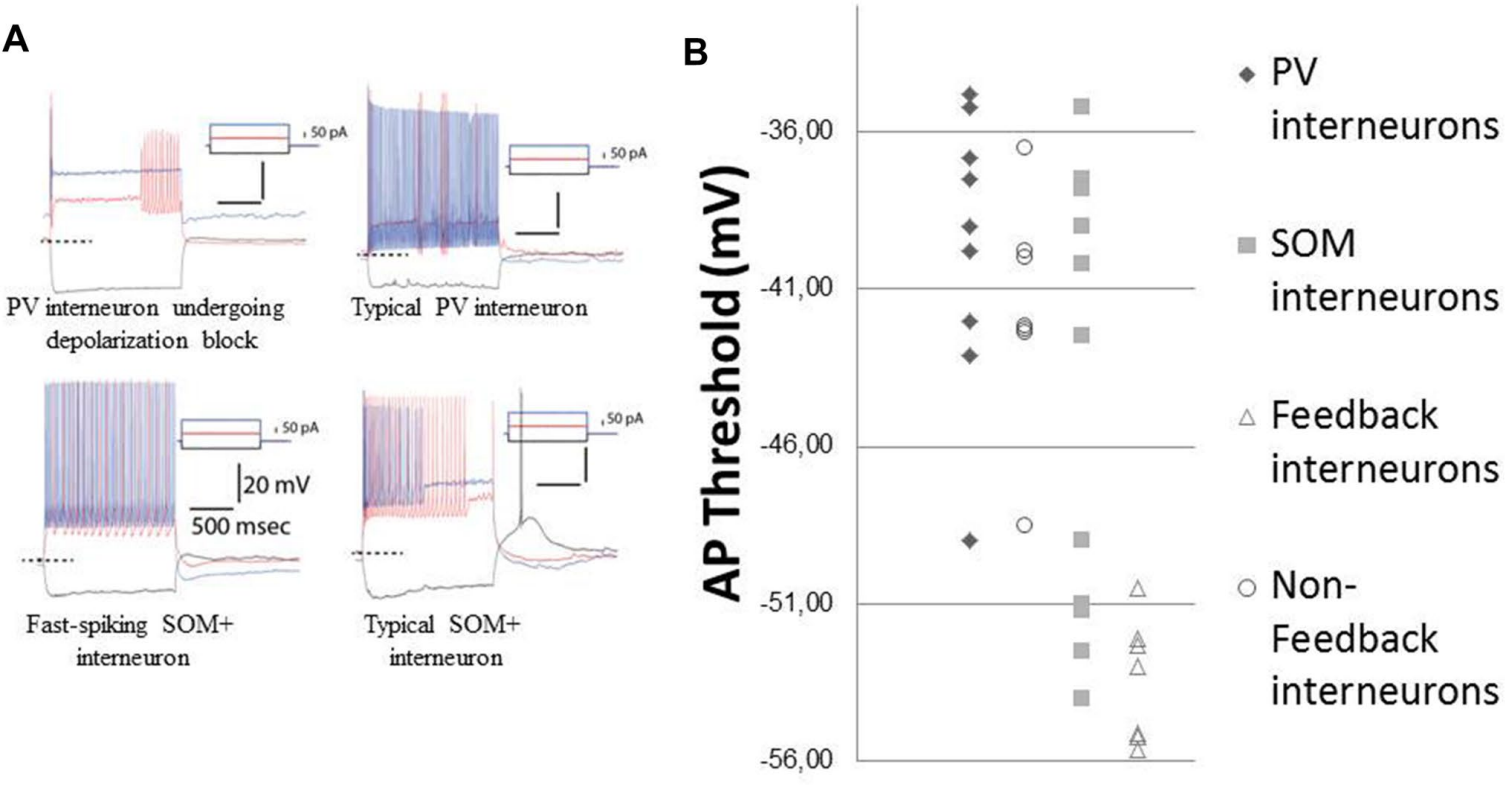
**a** Two parvalbumin (PV) (upper panel) and two somatostatin (SOM) (lower panel) interneurons with different electrophysiological profiles. The examples on the right sides represent the more typical electrophysiological profile for these populations. **b** Scatter plot of action potential thresholds for SOM and PV interneurons along with data from previous experiments

In our recordings, 6/9 PV interneurons (3 animals) could be regarded as classical fast spiking as evidenced by narrow spikes (0.9–1.2 ms) and high firing frequency (60–100 Hz) and they resembled the non-feedback interneurons from our recordings taken from wild type animals. The rest, exhibited spike frequency adaptation and some degree of depolarization block (Fig. 7a, top raw), in line with the heterogeneity observed in the BLA and other regions (Cauli et al. 2000; Rainnie et al. 2006; Woodruff and Sah 2007; Sosulina et al. 2010). Conversely, 4 SOM+ neurons out of 11 SOM+ neurons (four animals) exhibited a low action potential threshold comparable to the feedback LTS interneurons recorded in the previous experiment. In addition, these cells exhibited similarities with the LTS feedback interneurons in other respects such as the presence of spike frequency adaptation and a voltage sag indicative of an h-current. A minority (3/11) of SOM+ interneurons behaved like fast spiking interneurons (Fig. 7a, bottom raw).

**Fig. 8:**
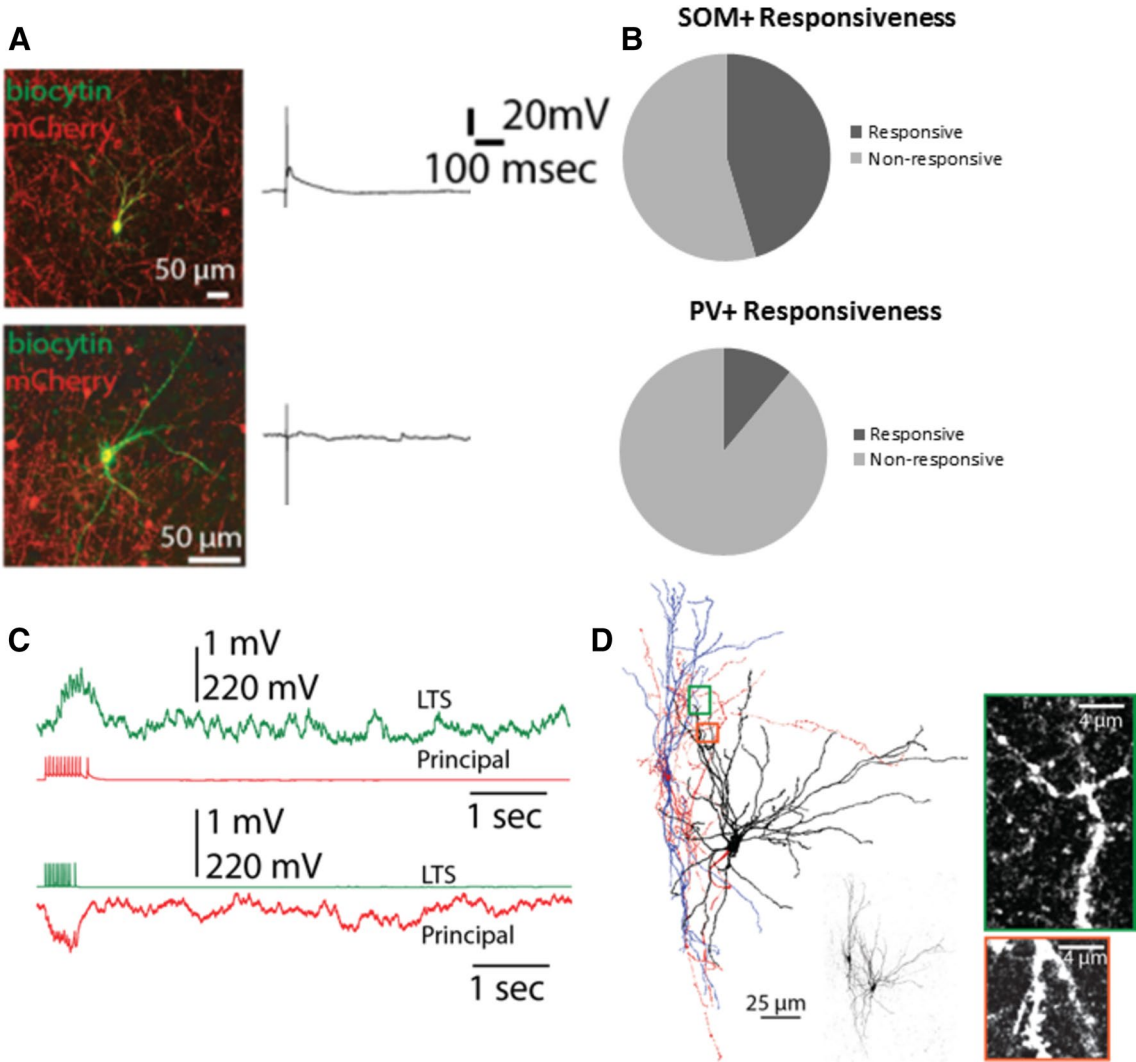
Evidence for the feedback interneuron function of somatostatin+ (SOM+) interneurons. **a** Representative responses of SOM+ (top) and parvalbumin+ (PV+) (bottom) neurons to BL stimulation. Micrographs are on the left and electrophysiological traces are on the right. **b** Bar charts illustrating responsiveness rates as evidenced by spiking activity to BL stimulation (5/11 in SOM + interneurons and 1/9 in PV interneurons). **c** Reciprocally connected SOM+ interneuron and an LA principal cell. **d** Morphological reconstruction of the recorded pair in C (left), original biocytin images of the entire neurons (middle) and magnified images of putative contact sites (right) highlighted on the reconstruction

When we electrically stimulated the BL using 100 μA currents, we observed spikes in 5/11 SOM+ interneurons (Fig. 8a, b, top). Interestingly, those interneurons were the ones with lowest action potential thresholds as revealed by an independent samples t-test between spiking versus non-spiking SOM+ interneurons [*t* (9) = 9.47, *p* < 0.0001]. We tested 3 out of these 5 BL stimulation responsive neurons utilizing paired recordings. In all of these cases, we found a presynaptic principal cell which also was innervated by the recorded SOM+ interneuron (Fig. 8c, d). Conversely, 1/9 PV+ interneurons exhibited spiking to BL stimulation. When we used higher current intensities (150 μA and above), PV+ interneurons exhibited small responses (< 5 mV). Unfortunately, we lost the BL-responsive PV+ interneuron before conducting paired recordings. Nonetheless, its responsiveness constitutes a strong evidence for the possibility that it receives inputs from principal LA neurons.

Table 1 summarizes the statistical comparisons of membrane properties (input resistance, action potential threshold, maximum firing frequency, adaptation ratio, and sag ratio) used in interneuron identification across different experiments. A one-way ANOVA has revealed a main effect on input resistance [*F* (3, 30) = 10.211, *p* < 0.000] and adaptation ratios [*F* (3, 30) = 6.082, *p* = 0.002]. LSD post hoc tests revealed that feedback interneurons had significantly higher input resistances from all other groups (*p* < 0.05 for all comparisons) in addition to a significant difference between SOM+ and PV+ interneurons (*p* = 0.046). LSD post hoc tests revealed also that the feedback interneurons exhibited a higher spike frequency adaptation as compared to all other groups (*p* < 0.05). The inability of ANOVA to capture similarities between feedback and SOM+ interneurons from different experiments likely signifies the fact that there is more than one type of SOM+ interneuron as in other brain regions. In line with this notion, when feedback interneurons were compared to BL-responsive SOM+ interneurons with independent samples t-tests, not a single difference emerged in any of the electrophysiological parameters reported while significant differences between BL-responsive SOM+ interneurons and BL-unresponsive SOM+ interneurons were observed along multiple dimensions including their input resistances (*p* < 0.01), adaptation ratios (*p* < 0.01), and action potential thresholds (*p* < 0.001) (see Table 1). Last but not the least, when we compared specifically the BL-responsive SOM+ interneurons to PV+ interneurons, significant differences emerged with regards to input resistance [*t* (12), 4.10, *p* < 0.01], adaptation ratio [*t* (12), 5.10, *p* < 0.001], sag ratio [*t* (12), 0.73, *p* < 0.05], and action potential thresholds [*t* (12), 5.41, *p* < 0.001] (see Table 1).

**Table 1:** Membrane properties of interneuron populations recorded

Interneuron type		Input Resistance (MΩ)		Action Potential Threshold (mV)		Maximum firing (Hz)		Adaptation ratio^d^		Sag ratio^e^	
Feedback Interneuron (7)		664.7 ± 73.6 ***^a^		− 53.4 ± 0.7		35.4 ± 5.9		3.11 ± 0.5***^b^		1.09 ± 0.04	
Non-feedback Interneuron (7)		301 ± 39.7		− 41.7 ± 1.3		51.0 ± 5.2		1.41 ± 0.1		1.03 ± 0.01	
SOM + Interneuron (11)		397.7 ± 57.9*^c^		− 44.5 ± 2.1		45.4 ± 4.2		2.14 ± 0.4		1.18 ± 0.10	
BL-responsive SOM + interneuron (5)	BL-Unresponsive SOM + interneuron (6)	552.4 ± 79.7	268.8 ± 27.8^##^	− 51.5 ± 0.8	− 38.7 ± 1.0^###^	38.6 ± 2.85	51.0 ± 6.82	3.21 ± 0.48	1.25 ± 0.28^##^	1.38 ± 0.19	1.01 ± 0.01
PV + Interneuron (9)		250 ± 33.6		-39.7 ± 1.5		48.8 ± 5.3		1.29 ± 0.1		1.01 ± 0.01	

Data are expressed as Mean ± SEM

***^a^, ***^b^Shows the marked groups significantly differing from all other groups (*p* < 0.005)

*^c^Marks a significant difference between SOM+ and PV+ interneurons. Moreover, ^#^ symbol indicates significant differences between BL-responsive and BL-unresponsive SOM+ interneurons with ^##^ indicating a *p* < 0.01 and ^###^ indicating a *p* < 0.001

^d^Interval of last two spikes/interval of first two spikes at half-maximal firing

^e^Largest decrease of voltage/steady state voltage after a current injection that brings the neuron’s steady state voltage nearest to − 90 mV during the *V*–*I* curve acquisition procedure

These results indicate that BL-responsive SOM+ interneurons (which are also feedback SOM+ interneurons) constitute a distinct population than BL-unresponsive SOM+ and PV+ interneurons.

## Discussion

The current study aimed to elucidate the mechanisms of intranuclear feedback inhibition within the LA. The largely unidirectional information flow within the BLA complex and the preservation of feedback loops in the same rostro-caudal plane (Samson et al. 2003) makes the coronal slices feasible for studying feedback inhibition using BL-stimulation and paired recordings. Indeed, feedback inhibition was evident both using BL-evoked responses in LA principal neurons and paired recordings. Paired recordings between principal neurons have shown that this inhibition form is not uncommon (15%) in this region.

We relied on certain parameters to identify the feedback interneuron in the LA circuitry. The first criterion entailed finding an interneuron that was reciprocally connected with the principal neurons as this is an identifying feature of feedback circuits. Secondly, we relied on the kinetic analysis of unitary IPSPs evoked by the interneurons in interneuron—> principal neuron pairs and polysynaptic IPSPs observed during principal neuron—> principal neuron recordings. Based on these, this study concludes that polysynaptic IPSPs are elicited by interneurons that have lower action potential thresholds (LTS interneurons). When we analyzed these polysynaptic IPSPs, we relied on the event detection tool in clampfit program to dissect only those events that had a rising phase that could be explained with a single exponential and a decay phase after the peak. It is critical to note that this might have introduced a degree of bias in data analysis and could have masked the visualization of IPSPs coming from other sources. The LTS interneurons exhibited a continuum of spike frequency adaptation and voltage sag indicative of h-current, making them an electrophysiologically distinct group from fast spiking cells. Experiments done in SOM-tdtomato and PV-tdtomato animals have revealed remarkable similarities between LTS and a proportion of SOM+ interneurons. These findings are in line with observations in other brain regions (Gibson et al. 1999; Beierlein et al. 2003; Goldberg et al. 2004). A limitation in our current approach entails the wide range in the distance among recorded pairs (10 to 200 microns). While it is possible that keeping a smaller distance could generate different results, this scenario is partially negated by our antidromic stimulation experiments which entails stimulation of proximal neurons as well.

Given our conditions that include the use of mature animals for slice experiments and a requirement for long-lasting recordings that are typically associated with intracellular dialysis, we could not couple biocytin fillings with post hoc immunocytochemistry (e.g. Kawaguchi and Kubota 1996; Kawaguchi and Kondo 2002) for determining the neurochemistry of LTS interneurons. A more fruitful approach proved to be using PV- and SOM- cre mice crossed with td-tomato lines to obtain targeted recordings from PV and SOM interneurons: This allowed us to make inferences about the neurochemistry of the interneuron involved in feedback inhibition. PV interneurons in the LA with one exception (1/9) did not possess feedback interneuron properties. On the other hand, SOM+ interneurons which exhibited electrophysiological similarities with LTS interneurons all exhibited spiking activity in response to BL stimulation suggesting that they receive local inputs from LA principal neurons. One pitfall in our interpretation concerns the lack of spikes in LTS interneurons during paired recordings as these neurons were recorded around a membrane potential of − 70 mV for consistency purposes. In this respect, experiments utilizing voltage sensitive dyes or calcium sensors are required for a more definitive conclusion. Nonetheless, it is important to re-iterate that LTS interneurons constituted the major cell type responsive to the excitation of principal neurons during paired recordings. These findings are in line with studies from cortex where LTS neurons constitute the SOM+ Martinotti cells serving vital functions in feedback inhibition (Kawaguchi and Kubota 1996; Goldberg et al. 2004; Wang et al. 2004; Ma et al. 2006). The absence of such responses in PV interneurons rules out the possibility of a potential contamination from the stimulation of extra-amygdalar inputs. Furthermore, SOM+ interneurons resembling LTS interneurons were reciprocally connected to principal neurons at a very high rate and the uIPSPs they evoked had slow rise times, consistent with the notion that these feedback interneurons are dendrite targeting interneurons (Muller et al. 2007; Fino and Yuste 2011). Last but not the least, the feedback LTS interneurons observed in our recordings from both wild type and SOM-tdtomato crosses received facilitating synapses from principal neurons, a recurring motif in other cortical regions where SOM+ Martinotti cells serve as feedback interneurons (Thomson and Dechars 1997; Markram et al. 1998; Wang et al. 2004).

A number of anatomical studies have found that PV+ interneurons receive inputs from local BLA principal neurons in different species while receiving minimal extra-amygdalar input (Smith et al. 2000; Woodruff and Sah 2007; Unal et al. 2014; Spampanato et al. 2016) while no direct data exists for SOM+ interneurons. These anatomical studies receive support by Woodruff and Sah (2007) who observed around 27% connectivity in the principal cell to PV+ interneuron direction in the BLA while we observed 1 out of 9 PV+ interneurons responsive to the activation of local LA circuitry as a result of BL stimulation. This suggests a lower portion of PV+ interneurons (11%) serve as feedback interneurons in the LA. The discrepancy with our study could be accounted by a number of factors either in isolation or interacting with each other. First of all, we do not have direct information about the neurochemistry of LTS feedback cells we recorded in wild type mice. A proportion of these cells could well be PV+ . A second interesting possibility pertains to subtle differences of the network architecture of LA and other BLA nuclei: we have exclusively recorded in LA neurons while the entire BLA was covered by Woodruff and Sah (2007). Our observation of PV immunoreactivity in SOM-tomato cells in the BL but not in the LA potentially signifies the different developmental history of PV and SOM interneurons in these structures. A third possibility pertains to the age of animals as Woodruff and Sah (2007) used younger (PD 16–25) mice while we used PD 30–60 mice.

Another factor pertaining to species differences could underline the discrepancy of the current results with anatomical studies (Smith et al. 2000; Unal et al. 2014). These studies point out to the notion that PV+ cells might not be contributing as much to feedforward inhibition while studies done in rats have shown that fast-spiking interneurons, which are typically PV+ in the BLA as well, constitute a responsive population to extra-amygdalar stimulation (Szinyei et al. 2000; Sosulina et al. 2010; Spampanato et al. 2011). It is important to note that, our experiments do not negate the possibility of SOM+ interneurons exerting feedforward inhibition (Unal et al. 2014) and other functions (McDonald et al. 2012; Capogna 2014) as is the case in the neocortex (Beierlein et al. 2003; Cruikshank et al. 2010).

The current study adds to the repertoire of potential functions, such as disinhibition (Wolff et al. 2014) and long-range projections (McDonald et al. 2012), for SOM+ interneurons. Feedback inhibition is a circuit mechanism thought to be critical for the formation of specific engrams within the amygdala (Kim et al. 2013; 2016). Furthermore, the dendritic location of feedback interneuron inputs on principal neurons elsewhere endows these neurons with the capability of regulating excitatory inputs to the level of single dendritic branches and even spines (Golding et al. 2002; Kampa et al. 2006; Humeau and Luthi 2007; Lovett-Barron et al. 2012; Bar Ilan et al. 2013; Kim et al. 2013, 2016; Cichon and Gan 2015) determining the exact locus of synaptic plasticity and formation of specific memories (Cichon and Gan 2015. In essence, the abovementioned mechanisms might be instrumental in parsing the LA into distinct sensory/functional compartments as is the case in other cortical regions (e.g. Fino and Yuste 2011; Adesnik et al. 2012; Zhang et al. 2014).

This study concludes that LTS interneurons which are predominantly SOM+ perform feedback inhibition functions in the LA. Further optogenetic studies are required to obtain data with higher throughput in the LA and other BLA nuclei along with functional studies testing their implications.
